# Successful Desensitization to mRNA COVID-19 Vaccine in a Case Series of Patients With a History of Anaphylaxis to the First Vaccine Dose

**DOI:** 10.3389/falgy.2022.825164

**Published:** 2022-02-02

**Authors:** Faisal AlMuhizi, Shaonie Ton-Leclerc, Michael Fein, Christos Tsoukas, Lene Heise Garvey, Derek Lee, Moshe Ben-Shoshan, Ghislaine A. C. Isabwe, Ana M. Copaescu

**Affiliations:** ^1^Division of Allergy and Clinical Immunology, Department of Medicine, McGill University Health Centre, McGill University, Montreal, QC, Canada; ^2^Department of Internal Medicine, Security Forces Hospital Program, Riyadh, Saudi Arabia; ^3^Faculty of Medicine, McGill University, Montreal, QC, Canada; ^4^The Research Institute of the McGill University Health Centre, McGill University, Montreal, QC, Canada; ^5^Allergy Clinic, Copenhagen University Hospital, Gentofte, Denmark; ^6^Department of Clinical Medicine, Copenhagen University, Copenhagen, Denmark; ^7^Pharmacy Department, Montreal General Hospital, Montreal, QC, Canada; ^8^Division of Pediatric Allergy and Clinical Immunology, Department of Medicine, McGill University Health Centre, Montreal, QC, Canada; ^9^Department of Infectious Diseases, Centre for Antibiotic Allergy and Research, Austin Health, Heidelberg, VIC, Australia

**Keywords:** vaccine, COVID-19, allergy, desensitization, challenge, polyethylene glycol, anaphylaxis

## Abstract

**Background:**

Coronavirus disease 2109 (COVID-19) vaccines have recently been approved to curb the global pandemic. The risk of allergic reactions to the vaccine polyethylene glycol (PEG) component has raised significant public concern. Desensitization is suggested in cases of vaccine related hypersensitivity reactions. After comprehensive literature review on the topic, our aim was to establish a safe and effective desensitization protocol for patients with suspected or confirmed immediate type hypersensitivity reactions to the COVID-19 vaccine.

**Methods:**

Participants were referred to the McGill University Health Center (MUHC) Allergy-Immunology department for clinical evaluation following a reported reaction to their first dose of Moderna® mRNA-1273 or Pfizer-BioNTech® BNT162b2 vaccines. They underwent skin prick testing (SPT) with higher and lower molecular weight (MW) PEG and polysorbate 80, as per published protocols. Their second dose was administered following a desensitization protocol consisting of multiple dose-administration steps followed by a 60-min observation period.

**Results:**

Among a cohort of 142 patients with an increased risk for allergic reactions to the COVID-19 vaccines, six individuals were selected to undergo desensitization. All were female with allergic background including chronic spontaneous urticaria, anaphylaxis to medications, and/or vaccines. The main symptom after their first dose was difficulty swallowing with lightheadedness or immediate urticaria, angioedema, and/or dizziness. Two patients had positive skin testing. One patient was on chronic antihistamines which resulted in an inconclusive PEG skin test and the skin testing was negative for the three other patients. During the desensitization, two patients reported cutaneous symptoms of an immediate reaction and were managed with antihistamines. One of these patients also complained of ear pressure and had a drop in her systolic blood pressure, treated with intravenous fluids.

**Conclusion:**

This study suggests that some individuals with an immediate-type hypersensitivity reaction to their first dose of mRNA COVID-19 vaccine may safely receive their second dose using a desensitization protocol. The success of this desensitization protocol is a step forward in the fight against COVID-19, allowing more individuals to be immunized.

## Introduction

A year and a half after the appearance of the severe acute respiratory syndrome coronavirus 2 (SARS-CoV-2) virus responsible for the coronavirus disease 2019 (COVID-19) pandemic, there have been more than 260 million cases of infection and over 5 million deaths worldwide (1). The emergence of approved vaccines and the efforts of mass vaccination have tremendously helped curb the progression of this pandemic. Among the approved vaccines, the Pfizer-BioNTech® BNT162b2 and the Moderna® mRNA-1273 vaccines have an innovative mechanism of action involving the use of a lipid nanoparticle-encapsulated mRNA encoding the spike protein of SARS-CoV-2. They both help protect against COVID-19 with a significant efficacy of 95 and 94.1%, respectively (2, 3). For both vaccines, allergic reactions and anaphylaxis remain rare and the mechanism has not been fully understood. At this stage, the rate of anaphylaxis is estimated at 11.1 per million doses for the Pfizer-BioNTech® vaccine and at 2.5 per million doses for the Moderna® vaccine (4). The majority of anaphylaxis cases are thought to be related to excipients in the vaccine, such as polyethylene glycol (PEG).

According to the current studies, two doses of a mRNA vaccine are required for adequate SARS-CoV-2 protection. However, receiving a second dose of vaccine represents a risk for individuals having a confirmed allergic reaction to the first dose. Administering the second dose in a controlled setting following a desensitization protocol can reduce this risk. Indeed, in a scenario where immunization is warranted and a patient has a confirmed reaction to a component of the vaccine, a graded-dose desensitization procedure is recommended (5). We report the successful desensitization in a cohort of six patients with reported immediate-type COVID-19 vaccine reactions.

## Methods

Participants were selected as a subgroup from the ongoing *Allergic Reactions to COVID-19 Vaccine* (ARCOV) study cohort, a 12-month prospective study aiming to evaluate the incidence of systemic and local allergic reactions to the COVID-19 vaccine in patients with previous history of severe allergic reactions. The selection criteria for the ARCOV study includes individuals referred to the McGill University Health Center (MUHC) outpatient allergy clinic who have a documented history of anaphylaxis, according to the *World Allergy Organization Anaphylaxis Guidance 2020* (6). Patients were excluded if they had an illness that would substantially increase the risk associated with their participation in the study, including but not limited to acute infectious illness, severe cardiac, respiratory disease or uncontrolled mastocytosis. From this cohort, participants having an anaphylaxis reaction after receiving their first dose of vaccine were selected to undergo desensitization. Selected patients underwent skin prick testing (SPT) for PEG and polysorbate 80.

Evaluated patients underwent PEG SPT with lower molecular weight (MW) PEGs: Polyoxyl 35 hydrogenated castor oil (Cremophor EL) (527 mg/ml), PEG 300 (100% wt./vol), PEG 3,000 (50% wt./vol), PEG 3,350 (50% wt./vol), polysorbate 80 (20% wt./vol), and high MW PEG 20,000 (0.01%, 0.1%, 1%, and 10% wt./vol) (7). Polyethylene glycol-derivatives were prepared at the laboratory of McGill University Research Institute, Montreal, Canada. The Cremophor EL (527 mg/ml) was purchased from Milipore Sigma Cremophor EL cat # 238470. As per published protocols, this product was diluted in ethanol 50%. Our specialized laboratory technician prepared the solution as one-part ethanol and one-part Cremophor EL. This solution was mixed well (vortex) until a clear solution was obtained. We performed SPT to PEG 3,350 50% wt./volume by diluting PEG 3,350 (Lax-A-Day, PegLyte or Emolax) in water (17 g in 34 ml). For the other included products, Macrogol/ PEG 300 (100% w/v) cat # 81162, Macrogol PEG 3,000 (50% w/v) cat # 8.19015.1000, polysorbate 80 (20% v/v) cat # P1754, and high MW Macrogol PEG 20,000 (0.01, 0.1, 1, and 10% w/v) cat # 813300, the dilutions were performed as per previously published protocol from Garvey et al. (7). These products were purchased from Milipore Sigma. Skin prick testing was performed on the forearm with a positive histamine 10 mg/ml control and a negative normal saline control. Previously published non-irritation concentrations were used except for Cremophor EL which was deemed non-irritant and negative on both immediate and delayed skin testing after being performed on five healthy participants. A positive reaction was defined as a wheal diameter of 3 mm or more from the negative control.

Patients received the Moderna® mRNA-1273 vaccine in four undiluted doses. The first two doses given were 0.05 ml each, while the following two doses were 0.2 ml each for a total of 0.5 ml. The Pfizer-BioNTech® vaccine, was administered in three undiluted doses: 0.05, 0.1 ml then 0.15 ml, for the total of 0.3 ml. Depending on their initial anxiety levels, the patients could be administered a placebo dose of 0.15 ml of normal saline injected intra-muscular, as per our current assessment protocols. Depending on their initial reaction to this placebo dose, they could receive 1–2 subsequent placebo doses before the vaccine administration. The doses were administered at 20 min intervals and followed by a 60-min observation after the last dose. Please refer to Tables 1A,B for the desensitization protocols.

**Table 1A:** Desensitization protocol used for administration of a second dose of Moderna® mRNA-1273 in patients having experienced an immediate type of allergic reaction following administration of their first dose.

**Step**	**Dose**	**Cumulative**	**Dilution**	**Cumulative**
	**(ml)**	**dose (ml)**		**time (time)**
^*^P	0.15	n/a		0
1	0.05	0.05	Full strength	20
2	0.05	0.1	Full strength	40
3	0.2	0.3	Full strength	60
4	0.2	0.5	Full strength	80
	Observation			140

* *Patients were administered a placebo dose, consistent of normal saline injected intra-muscular, based on their anxiety level upon arrival at the allergy clinic, as per local hospital protocol*.

**Table 1B:** Desensitization protocol used for administration of a second dose of Pfizer-BioNTech® in patients having experienced an immediate type of allergic reaction following administration of their first dose.

**Step**	**Dose**	**Cumulative**	**Dilution**	**Cumulative time (time)**
	**(ml)**	**dose (ml)**		**time (time)**
^*^P	0.15	n/a		0
1	0.05	0.05	Full strength	20
2	0.1	0.15	Full strength	40
3	0.15	0.3	Full strength	60
	Observation			120

* *Patients were administered a placebo dose, consistent of normal saline injected intra-muscular, based on their anxiety level upon arrival at the allergy clinic, as per local hospital protocol*.

The study was approved by the McGill University Health Center Research Ethics Board (REB# ARCOV/2021-7510). All participants included provided a written informed consent.

## Results

Six participants from the ongoing recruited ARCOV cohort of 142 patients met the inclusion criteria and underwent desensitization (Table 2). All patients were female. Three of the patients were Caucasian with the other three being of Aboriginal, Hispanic, and East Asian origin. Five patients had a significant past medical history of an allergic reactions to either a drug or a vaccine. Half of the patients reported an immediate reaction to the Moderna® mRNA-1273 vaccine and the other half to the Pfizer-BioNTech® vaccine. The patients received various treatments for their reactions: antihistamines (patient 1 and 2); IM epinephrine, PO prednisone, IV diphenhydramine, and PO ranitidine (patient 3); IV methylprednisolone succinate and IV diphenhydramine (patient 4); and six IM epinephrine injections and IV methylprednisolone succinate (patient 6). Patient 5 did not seek medical attention following her reaction. Please refer to the Supplementary Section 1 for a detailed individual case description and Table 2 for a summary description of these cases.

**Table 2:** Patient demographics, reaction to first vaccine dose, treatment of reaction, latency period between first dose, and allergist evaluation, result of PEG SPT and response to desensitization.

**Patient**	**Gender Age**	**Ethnicity**	**Comorbidities**	**Allergy labels**	**Reported symptoms first vaccine dose**	**PEG SPT result**	**Vaccine type**	**Desensitization**
1	F 49	Aboriginal	Anemia Neuropathic pain	Amoxicillin Cefazolin Azithromycin Shellfish	**15 min:** generalized erythema, urticarial, and dyspnea	Delayed positive (3 h) Cremophor EL	mRNA (Moderna)	First dose: itchiness Second dose: increased itchiness (no hives) Treatment: cetirizine 20 mg PO Fourth dose: cough and globus sensation
2	F 66	East Asian	Steroid-dependent spondylarthritis Colitis	Pneumococcal conjugate vaccine Recombinant zoster vaccine Anileridine Infliximab	**30 min:** generalized urticaria 24 h: angioedema eyes and lips	Delayed positive (5 h) Cemophor EL PEG 300 PEG 3000 PEG 3350	mRNA (Moderna)	Premedication: Rupatadine 20 mg PO Prednisone 10 mg PO
3	F 64	Caucasian	CSU Diverticulosis Hypothyroidism Oral lichen planus	Rabies vaccine	**15 min**: dizziness, itchiness of her feet, hands, arms, and throat with visible urticaria	Negative Histamine –Negative	mRNA (Moderna)	Premedication: Bilastine 40 mg. Third dose: headache Treatment: Acetaminophen
4	F 35	Caucasian	None	Penicillin Cephalosporin	**15 min**: throat itchiness and difficulty swallowing, nausea, vomiting, and lightheaded	Negative	mRNA (Pfizer-BioNTech)	Third dose: itchy arms and right ear pressure. 120 min after third dose: dizziness and drop in systolic BP Treatment: cetirizine 20 mg and IV fluid bolus
5	F 57	Caucasian	Bronchial Asthma Ehlers-Danlos syndrome Osteopenia	Septra	**20 min**: facial numbness and swelling, cough and difficulty breathing, hoarseness, and chest pain	Negative	mRNA (Pfizer-BioNTech)	Third dose: tongue tingling. 1 week: maculopapular rash, desquamation of the skin. Treatment: desloratadine 10 mg PO, mometasone cream topical application.
6	F 33	Hispanic	Asthma Depression Post-traumatic stress disorder	Codeine Shellfish Peanut	**10 min**: throat itchiness and swelling, wheezing, vomiting, generalized numbness	Negative	mRNA (Pfizer-BioNTech)	Premedication: Cetirizine 40 mg PO Placebo: neck itchiness First dose: muscle spasms

*BP, blood pressure; CSU, chronic spontaneous urticaria; F, female; PO, by mouth*.

All the patients were assessed at the MUHC outpatient allergy clinic more than 2 months after their initial reported reaction and had PEG SPT performed. Patient 1 had a negative immediate SPT but a delayed positive reaction to Cremophor EL (3 h). Patient 2 had a delayed positive response (5 h) to Cremophor EL, PEG 300, PEG 3,000, and PEG 3,350. This patient had also reported an allergic reaction to infliximab previously, an agent that contains polysorbate 80. Her skin testing was negative to polysorbate 80, which indicate her previous reaction to infliximab was not caused by this excipient. The other patients had negative PEG skin testing, however patient 3 had a faintly positive histamine control as she was taking antihistamines during the evaluation.

Prior to the desensitization protocol, three patients were premedicated with antihistamines: patient 2 received rupatadine 20 mg PO, patient 3 received bilastine 40 mg PO, and patient 6 was administered cetirizine 40 mg PO. The other patients did not receive any premedication. Three of them received their second dose of Moderna® mRNA-1273 vaccine and three received their second dose of Pfizer-BioNTech® vaccine. All the patients that received the Pfizer-BioNTech® vaccine and patient 1 (received Moderna® mRNA-1273 vaccine) were also administered a placebo. Patient 6 reported neck pruritus after the placebo dose but subsequently tolerated well the vaccine. Mild reactions were reported during the desensitization protocol: pruritus (patient 1 and 6), globus sensation (patient 1), headache (patient 3), tingling sensation in the tongue (patient 5), and muscle spasms (patient 6). Patient 4 reported pruritus and redness over both her hands and arms as well a pressure in the right ear and was treated with cetirizine 20 mg PO and her symptoms improved within 30 min. She also presented a decrease in her systolic blood pressure and pulse, consistent with a possible vasovagal reaction, that was treated with intravenous fluids.

**Figure 1:**
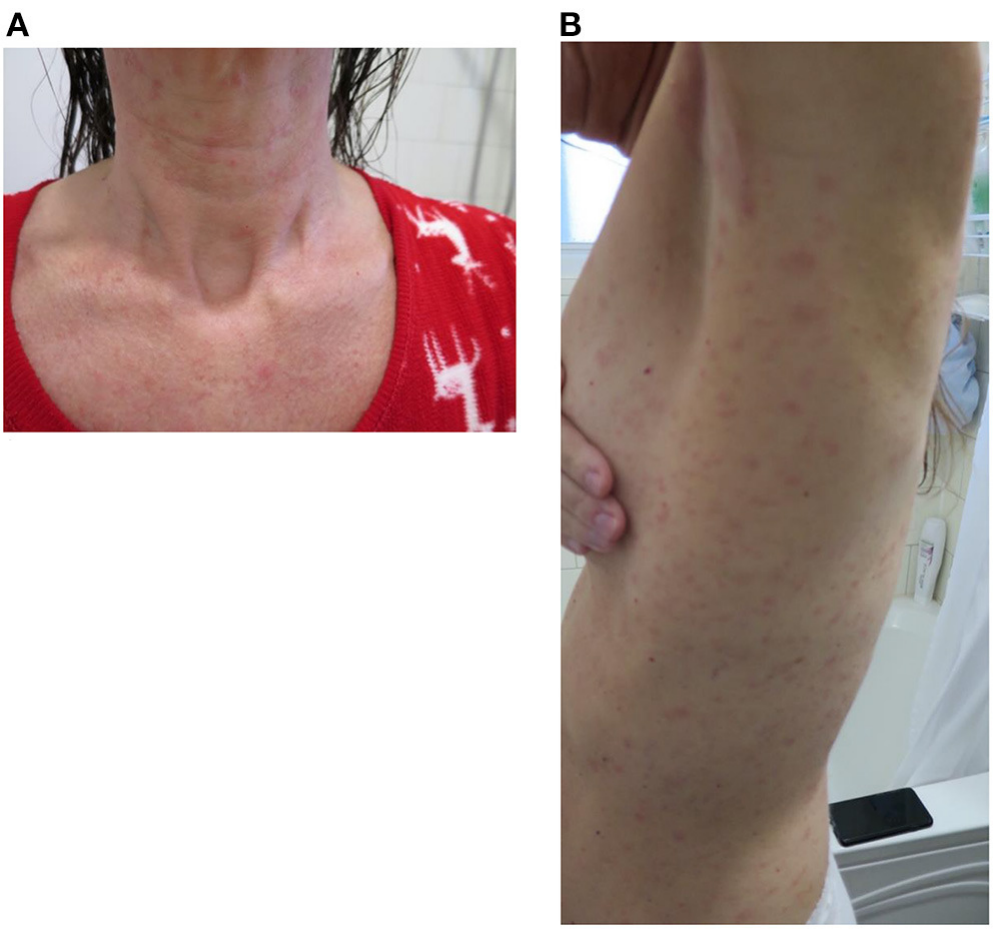
Patient 5 reaction to vaccination: generalized erythematous maculo-papular reaction **(A)** front cervical view and **(B)** later trunk view.

When contacted 1 week after their dose, the majority of the patients reported mild adverse reactions. Patient 2 reported mild pruritus that started 3 days after the desensitization and was well-controlled with cetirizine. Patient 3 described hives on the third and fourth day post-desensitization which were controlled with bilastine. Patient 4 had persistent fatigue following the vaccine dose as well as recurrent headache despite acetaminophen regular use. Patient 5 developed a whole-body generalized maculopapular rash (Figure 1) that was managed with PO desloratadine and mometasone topical cream. This skin eruption lasted for 3 weeks and was accompanied by a mild skin desquamation but no severity criteria. Patient 6 reported recurrent cough and throat closure sensation that was managed well with asthma puffers at home.

## Discussion

This study suggests that individuals who present with an immediate-type hypersensitivity reaction to their first dose of COVID-19 vaccine may safely and successfully be immunized with their second dose using a desensitization protocol. All participants that underwent desensitization had a prior history of drug and/or vaccine hypersensitivity. Moreover, patient 3's past medical history was significant for chronic spontaneous urticaria. Hence, the hives she experienced on the third and fourth day following the desensitization process could have been due to an exacerbation of her previous condition and not the vaccination itself. Patient 5 presented a delayed non-severe generalized maculo-papular eruption. Various dermatological conditions have been reported in the literature following SARS-CoV-2 vaccination with morbilliform eruptions occurring in up to 7% of patients (8).

### Vaccine Desensitization Protocols

Successful desensitization to vaccines including diphtheria, tetanus, and pertussis (DPT) vaccine, the measles, mumps, and rubella (MMR) vaccine, the yellow fever vaccine, the various influenza vaccines have been previously reported, and more recently a successful desensitization to the Pfizer-BioNTech® BNT162b2 vaccine (Table 3) (5, 9–16).

**Table 3:** Desensitization protocols suggested for influenza, measles, tetanus, MMR, DPT, yellow fever, and Pfizer-BioNTech® from 1990 to 2021.

**Vaccine**	**Step**	**Dose (ml)**	**Dilution**	**Time (min)**
Influenza (9)	1	0.05	Full strength	q30
	2	0.1	Full strength	
	3	0.15	Full strength	
	4	0.2	Full strength	
Influenza (10)	1	0.05	1:100	q15
	2	0.05	1:10	
	3	0.05	Full strength	
	4	0.10	Full strength	
	5	0.15	Full strength	
	6	0.20	Full strength	
H1N1 Influenza (11)	1	0.05	1:10	q15
	2	0.05	Full strength	
	3	0.10	Full strength	
	4	0.15	Full strength	
	5	0.20	Full strength	
Influenza (12)	1	0.05	1:1,000,000	q30
	2	0.05	1:100,000	
	3	0.05	1:10,000	
	4	0.05	1:1,000	
	5	0.05	1:100	
	6	0.05	1:10	
	7	0.05	Full strength	
	8	0.05	Full strength	
Pfizer BioNTech (5)	1	0.03	Full strength	q30
	2	0.07	Full strength	
	3	0.10	Full strength	
	4	0.10	Full strength	
Measles (10)	1	0.05	1:100	q15
	2	0.05	1:10	
	3	0.05	Full strength	
	4	0.05	Full strength	
	5	0.05	Full strength	
	6	0.05	Full strength	
	7	0.05	Full strength	
	8	0.05	Full strength	
	9	0.05	Full strength	
	10	0.05	Full strength	
	11	0.05	Full strength	
	12	0.05	Full strength	
Measles, Mumps, and Rubella (13)	1	0.05	1:100	q20
	2	0.5	1:10	
	3	0.05	Full strength	
	4	0.10	Full strength	
	5	0.15	Full strength	
	6	0.20	Full strength	
Tetanus (14)	1	0.02	1:1,000	q20
	2	0.02	1:100	
	3	0.02	1:100	
	4	0.02	1:10	
	5	0.10	1:10	
	6	0.05	Full strength	
	7	0.10	Full strength	
	8	0.15	Full strength	
	9	0.20	Full strength	
Toxoid-containing vaccines (tetanus, diphtheria) (15)	1	0.05	1:100	q20
	2	0.05	1:10	
	3	0.05	Full strength	
	4	0.10	Full strength	
	5	0.20	Full strength	
	6	0.25	Full strength	
Yellow Fever (16)	1	0.1	1:1,000	q30
	2	0.1	1:100	
	3	0.1	1:10	
	4	0.05	Full strength	
	5	0.1	Full strength	
	6	0.15	Full strength	
	7	0.2	Full strength	
Suggested universal desensitization protocol (17)	1	0.05	1:10	q15–20
	2	0.05	Full strength	
	3	0.10	Full strength	
	4	0.15	Full strength	
	5	0.20	Full strength	

All desensitization protocols have between 4 and 12 steps with doses being administered every 15–30 min depending on the vaccine. This was the case with our Moderna® mRNA-1273 vaccine desensitization procedure as well (Table 1A). The Pfizer-BioNTech® BNT162b2 vaccine was administered in three steps following multidisciplinary discussion with the nursing and pharmacy team. Indeed, the volume required for a dose below 0.05 ml would pose a technical issue and would be difficult to inject using a hypodermic needle. Most protocols also suggest the administration of a diluted dose before moving on the full-strength doses (Table 3). The dose volume administered varies between 0.02 and 0.2 ml, usually progressively increasing the volume or strength with each subsequent dose. The first dose is always the most diluted and the concentration gradually builds up to full strength (Table 3). Dose dilution varies from 1:100 to 1:10 for most vaccines, but the first dose may be as diluted as 1: 1,000,000 as for the influenza vaccine desensitization performed in 2018 (9). Although there are some similarities, protocol variations depend on multiple factors including the degree of hypersensitivity of the patient's undergoing desensitization. In 2003, there was a suggested universal vaccine desensitization protocol consisting of five steps, gradually increasing the dose volume and the strength: the first dose being diluted and the other four being delivered at full strength every 15–20 min (17). However, in the context of a global pandemic, in both the Moderna® mRNA-1273 and the Pfizer-BioNTech® BNT162b2 desensitization, dilution was not an option as it would mean discarding a dose of vaccine for dilution purposes. Furthermore, it is not known whether diluting these nanoparticle mRNA-based vaccines would jeopardize their stability and efficacy. The desensitization protocol performed for our patients was based on the influenza vaccine desensitization by Fung et al. (14) because it did not require administration of diluted doses. This influenza vaccine desensitization consisted of four doses of progressively increasing volume, administered at full strength at 30 min intervals.

The main differences between this study and the previously mentioned study on Pfizer-BioNTech® BNT162b2 desensitization lies in the selection criteria. Only one individual with a positive SPT to polysorbate 80 was desensitized, as they opted not to vaccinate those having a positive SPT to PEG (5). In our study, we desensitized one patient (#2) that had delayed-positive PEG testing.

### PEG and Polysorbate 80 Skin Prick Testing

In most cases of hypersensitivity reactions to vaccines, it is the excipients rather than the active ingredients that are the culprit. When it comes to the Pfizer-BioNTech® and Moderna® vaccines, the ingredient suspected to be responsible for the allergic reaction is PEG, although there is no solid evidence. Polyethylene glycol is an excipient that improves water solubility. It is also found in other drugs and cosmetic products. Polysorbate 80 is a compound which shares a similar structure to PEG and is used as an excipient in other vaccines; among these are included the AstraZeneca and Johnson & Johnson vaccines protecting against COVID-19. Although the two mRNA-vaccines are the first vaccines to contain PEG, it is proposed that first-dose hypersensitivity reactions could be due to previous sensitization to polysorbate 80 and cross-reactivity between polysorbate 80 and PEG (18). Despite current clinical guidelines contraindicating vaccination against SARS-CoV-2 in patients having an immediate hypersensitivity type reaction to PEG or polysorbate 80, it was recently suggested that excipient skin testing should not have a role in assessing possible reactions to these vaccines (19). In a cohort of 80 patients, 20% had positive SPT to PEG, polysorbate 80 or both (20). Indeed, 70 of them received their second dose and 89% had no symptoms or only mild symptoms managed with antihistamines. This suggests that excipient SPT in the context of SARS-CoV-2 vaccines has a poor sensitivity and does not adequately predict second dose tolerance. However, the poor specificity is very likely to be related to the use of intradermal testing for polysorbate 80 using a product with an irritant effect (lubricant eye drops Refresh Tears?) (19). Apart from PEG, another component of the vaccine, 1,2-distearoyl-sn-glycero-3-phosphocholine (DSPC), should also be considered as a potential culprit as it contains a quaternary ammonium (QA) group. Patient 6 was allergic to codeine, a drug containing QA and her skin testing to PEG and polysorbate 80 resulted negative, suggesting that maybe PEG and polysorbate 80 could not be the only responsible agents for hypersensitivity reactions to SARS-2 vaccines (21).

### Tolerance of Second COVID-19 Vaccine Dose

In a recent meta-analysis describing patients with a reaction to first COVID-19 vaccine dose, data showed that administering the second dose by single or two steps is equally safe, especially in patients with mild reactions (4). Similar, a recent American multi-center retrospective study reported that many patients may tolerate a second dose of vaccine without desensitization despite reacting to their first dose (22). In a cohort of 189 patients having had reactions to their first dose, 159 received their second dose (22). All the patients tolerated this second dose with either no symptoms or mild symptoms which were self-limited or resolved with antihistamines. The authors proposed two hypotheses to explain this: (1) either the majority of these initial reactions did not have a true allergic origin or (2) they were allergic reactions but involved a non-IgE-mediated mechanism (22). The risk assessment based on a detailed history and skin testing with PEG and other drug excipients has allowed safe revaccination in other reported cohorts where 95% (52/55) of patients presenting adverse reactions to their first mRNA vaccine dose were safely revaccinated (23). The absence of an IgE-mediated mechanism is also underlined in other similar studies were patients with suspected first dose anaphylaxis had negative PEG testing and subsequently tolerated the second mRNA vaccine dose (23, 24).

Other strategies aiming to improve vaccination safety have been reported in the literature. The notion that PEG allergic patients can safely receive a non-PEG COVID-19 vaccine such as AstraZeneca has been described in the literature (25), even for patient with positive skin testing for polysorbate 80 (26). Further, a single dose omalizumab was been used as a pre-treatment option for two patients having presented an anaphylactoid reaction following their first mRNA COVID-19 vaccine dose (27). By performing placebo-controlled desensitization protocols for some of our patients, we aimed to reduce the nocebo effect (negative expectations of the patient regarding a treatment) that might be experienced by patients that presented with reactions after their first vaccine doses. Despite our desensitization process taking place prior to the above-mentioned publications, these articles provide a new outlook on our results. In this context, a detailed risk assessment evidence-based evaluation will be undertaken prior to the administration of a possible third dose or a booster dose.

In our cohort of patients referred for COVID-19 vaccine reactions, we identified six patients that presented objective immediate symptoms following their first vaccine doses. In all these cases desensitization was successfully performed. One of the main limitations to this study is that the rate of true allergic reaction to COVID-19 vaccines is very low, hence limiting recruitment of a large sample size. While four or six patients did receive placebo prior to their desensitization, placebo was not administered to all the included patients. The allergic reactions reported by the patients were limited to throat symptoms, urticaria, erythema, swelling, and dizziness, thus we don't know if this procedure would be successful for patients presenting with more severe anaphylaxis symptoms such as hypotension or respiratory compromise. Our study does not include a comparison group where the vaccines could be administered using single or two step challenge. In the context of a possible anaphylaxis, safety was a priority in spite of the time consuming and resource intensive requirements of a desensitization protocol. Despite great progress in our understanding of excipient skin testing and vaccine challenges, future studies are needed to compare second dose tolerance with and without the use of a desensitization protocol, following an individual risk stratification with a detailed history and excipient skin testing.

## Conclusion

Ensuring complete immunization is beneficial both for individual protection and herd immunity. Indeed, the partial immunity conferred by a single dose of mRNA vaccine could increase the risk of emergence of vaccine-resistant variants of SARS-COV-2. Moreover, immunization with two doses ensures long term protection against the virus (28). Current FDA recommendations for both the Pfizer-BioNTech® and the Moderna® vaccines state that only people who have had a severe allergic reaction to a previous dose of the vaccine or a severe allergic reaction to any ingredient of the vaccine should not receive the vaccine (29, 30). Individual risk assessment and optimal selection strategy for re-vaccination is imperative and vaccine desensitization is an important strategy for those having presented anaphylaxis following vaccine administration. The success of this desensitization protocol is a step forward in the fight against COVID-19: not only can it help reduce vaccine hesitancy related to possible allergic reactions, but it also gives the opportunity to immunize more individuals in a safe and structured manner, hence contributing to the efforts of mass vaccination.

## Data Availability Statement

The original contributions presented in the study are included in the article/Supplementary Material, further inquiries can be directed to the corresponding author/s.

## Ethics Statement

The studies involving human participants were reviewed and approved by McGill University Health Center Research Ethics Board (REB# ARCOV/2021-7510). The patients/participants provided their written informed consent to participate in this study. Written informed consent was obtained from the individual(s) for the publication of any potentially identifiable images or data included in this article.

## Author Contributions

ST-L performed the literature review and wrote the manuscript text with supervision from FA and AC. FA, MF, GI, and AC recruited patients, contributed to the desensitization protocol, and performed the desensitization. All authors reviewed the manuscript and made a substantial, direct, and intellectual contribution to the work. All authors approved the manuscript for publication.

## Funding

AC received support from the Montreal General Hospital Foundation and Research Institute of the McGill University Health Center (RI-MUHC).

## Conflict of Interest

The authors declare that the research was conducted in the absence of any commercial or financial relationships that could be construed as a potential conflict of interest.

## Publisher's Note

All claims expressed in this article are solely those of the authors and do not necessarily represent those of their affiliated organizations, or those of the publisher, the editors and the reviewers. Any product that may be evaluated in this article, or claim that may be made by its manufacturer, is not guaranteed or endorsed by the publisher.
