# Supplementary material for: Successful Desensitization to mRNA COVID-19 Vaccine in a Case Series of Patients With a History of Anaphylaxis to the First Vaccine Dose
Source: Front Allergy. 2022 Feb 2;3:825164. doi: 10.3389/falgy.2022.825164 (PMC8974752; doi:10.3389/falgy.2022.825164)
Supplement: Supplementary file 1 [file Data_Sheet_1.docx]

Supplementary Material

# Supplementary Data – Patient detailed information

**Patient 1** is a 49-year-old, Aboriginal woman known for anemia and neuropathic pain. Her allergic background was significant for allergy to azithromycin, cefazolin, amoxicillin, and shellfish. Upon receiving her first dose of the Moderna® vaccine, she reported immediate (less than 15 minutes) generalized erythema, urticaria and dyspnea. She was treated with cetirizine 10 mg PO. Two months later, she was assessed at the MUHC outpatient allergy clinic. She had a delayed positive reaction to Cremophor EL 3 hours after her SPT and negative testing for all the other agents evaluated. She underwent desensitization to the Moderna® mRNA-1273 vaccine without any premedication. She did not report any reaction after placebo administration. During the first 0.05 ml vaccine dose, she felt subjective pruritus. She was reassured and easily distracted to stop scratching. The pruritus worsened after the second 0.05 ml, so she was given cetirizine 20 mg. Her symptoms improved after 15 minutes, and she received the 3^rd^ and 4^th^ doses. After the final dose, she had a cough and globus sensation. The throat examination was completely normal with no signs of erythema or swelling. She was given water and her symptoms improved. She reported no other adverse reactions at the follow-up call a week later.

**Patient 2** is a 66-year-old woman of East Asian decent known for steroid-dependent spondylarthritis and colitis. Her allergic background was significant for seasonal allergic rhinitis, 1-2 episodes of urticaria per year and allergic reactions to the pneumococcal conjugate vaccine, recombinant zoster vaccine, anileridine and infliximab. Despite taking diphenhydramine and cetirizine before her first Moderna® vaccine dose, she presented with diffuse urticaria 30 minutes after her dose. She had also reported delayed (more than 24 hours) swelling of the eyes, throat, and lips that responded well to antihistamines. Two months after the reaction, she was clinically evaluated at the allergy clinic. She had a delayed positive response to Cremophor EL, PEG 300, PEG 3,000, and PEG 3,350, 5 hours after her SPT. She was premedicated with rupatadine 20 mg PO and underwent an uneventful desensitization to 2^nd^ dose of the Moderna® mRNA-1273 vaccine with a prolonged observation period of 120 minutes. When she was contacted a week after the vaccine, she reported pruritus that started 3 days after the desensitization that was well controlled with cetirizine.

**Patient 3** is a 64-year-old Caucasian woman who was known for diverticulosis, hypothyroidism, and oral lichen planus. Her allergic background was significant for asthma, chronic spontaneous urticaria and anaphylaxis to the rabies vaccine. After receiving her first dose of Moderna® mRNA-1273 vaccine, she presented with pruritus of her feet, hands, arms, and throat with visible urticaria and dizziness. She was treated with IM epinephrine, PO prednisone, IV diphenhydramine and PO ranitidine. Two months later, she was evaluated at the allergy clinic. The patient’s PEG SPT was negative, but the histamine control was only faintly positive as she was taking regular antihistamines. She was premedicated with bilastine 40 mg PO and her desensitization to the 2^nd^ dose of the Moderna® mRNA-1273 vaccine was uneventful. She reported a headache after the 3^rd^ dose for which she was administered acetaminophen. When she was contacted a week later, she reported hives on the 3^rd^ and 4^th^ day post-desensitization which were controlled with bilastine.

**Patient 4** is a 35-years-old Caucasian woman known for allergic rhinitis and penicillin/cephalosporin allergy. Fifteen minutes after receiving her 1^st^ dose of the Pfizer-BioNTech® vaccine, she reported throat pruritus and difficulty swallowing. Her vital signs were normal, and she was transferred to the emergency department. While waiting in the emergency room, she developed nausea, vomiting and lightheadedness. On exam, urticaria was observed on her chest. She was managed with IV methylprednisolone succinate and IV diphenhydramine. She was evaluated in allergy clinic and the PEG SPT was negative. During desensitization procedure, she did not report any reaction following the administration of 2 placebo doses. Ten minutes after her third divided dose of Pfizer-BioNTech® vaccine, she reported pruritus and redness over both her hands and arms as well a pressure in the right ear. She was treated with cetirizine 20 mg PO and her symptoms improved within 30 minutes. Two hours after the last dose while comfortably sitting on the examination chair, she reported pressure in her ears, lightheadedness and her systolic blood pressure dropped from 122 to 101, with her pulse dropping from 87 to 68, consistent with a possible vasovagal reaction. She was managed with 250 ml IV of normal saline with symptom improvement. When contacted the following week, the patient reported persistent fatigue following the vaccine dose as well as recurrent headache despite acetaminophen regular use.

**Patient 5** is a 57-year-old Caucasian woman known for asthma, Ehlers Danlos syndrome and osteopenia. She had reported facial numbness and swelling, cough and difficulty breathing, hoarseness and chest pain 20 minutes after receiving her 1^st^ Pfizer-BioNTech® vaccine. She did not seek medical attention and her symptoms persisted for several hours. Four months later, she was evaluated at the allergy clinic and PEG SPT was negative. Considering the reported reaction, desensitization to Pfizer-BioNTech® vaccine was performed. She did not report any reaction following the placebo dose administration. Fifteen minutes after the last vaccine dose, she reported tingling sensation in the tongue. There were no other objective findings at that time. Three days after her vaccination, she developed a whole-body generalized maculopapular rash (**Figure 1**) that was managed with PO desloratadine and mometasone topical cream. This skin eruption lasted for 3 weeks and was accompanied by a mild skin desquamation but no severity criteria.

**Patient 6** is a 33-year-old Hispanic woman known for asthma, depression, and post-traumatic stress She had reported allergic reactions to codeine, peanuts, and shellfish. Ten minutes after receiving the first dose of the Pfizer-BioNTech® vaccine, she reported throat pruritus and swelling, wheezing, vomiting as well as generalized numbness. She was managed in the vaccination center with 3 injections of IM epinephrine. As she was transferred to the emergency department, she was administered 2 other IM epinephrine doses. Further, at her arrival at the hospital, she was administered a 6^th^ IM injection of epinephrine as well as IV methylprednisolone succinate for ongoing symptoms. She was evaluated in the allergy clinic 3 months after the reaction and PEG SPT was negative. The patient was premedicated with cetirizine 40 mg PO, she was administered a placebo dose of 0.15 ml normal saline followed by the 3 doses of Pfizer-BioNTech® vaccine. After the placebo dose, she reported neck pruritus. Following the 1^st^ vaccine dose, she reported muscle spasms as well as persistent itchiness on her neck area. The patient completed the desensitization protocol and was observed for 60 minutes with no other reactions. At her one-week telephone follow-up, she reported recurrent cough and throat closure sensation that was managed well with asthma puffers at home.
